# MDM2 Implications for Potential Molecular Pathogenic Therapies of Soft-Tissue Tumors

**DOI:** 10.3390/jcm12113638

**Published:** 2023-05-24

**Authors:** Sylvia Yao Sun

**Affiliations:** Sarcoma Biology Laboratory, Department of Surgery, Memorial Sloan Kettering Cancer Center, 417 E 618 St, New York, NY 10065, USA; sy1viaysun5@gmail.com

**Keywords:** MDM2, soft-tissue tumors

## Abstract

Murine double minute 2 (MDM2, gene name *MDM2*) is an oncogene that mainly codes for a protein that acts as an E3 ubiquitin ligase, which targets the tumor suppressor protein p53 for degradation. Overexpression of MDM2 regulates the p53 protein levels by binding to it and promoting its degradation by the 26S proteasome. This leads to the inhibition of p53’s ability to regulate cell cycle progression and apoptosis, allowing for uncontrolled cell growth, and can contribute to the development of soft-tissue tumors. The application of cellular stress leads to changes in the binding of MDM2 to p53, which prevents MDM2 from degrading p53. This results in an increase in p53 levels, which triggers either cell cycle arrest or apoptosis. Inhibiting the function of MDM2 has been identified as a potential therapeutic strategy for treating these types of tumors. By blocking the activity of MDM2, p53 function can be restored, potentially leading to tumor cell death and inhibiting the growth of tumors. However, further research is needed to fully understand the implications of MDM2 inhibition for the treatment of soft-tissue tumors and to determine the safety and efficacy of these therapies in clinical trials. An overview of key milestones and potential uses of MDM2 research is presented in this review.

## 1. Introduction

The *MDM2* gene was first discovered as a gene responsible for the transformation of an immortalized mouse cell line, BALB/c 3T3, into a cancerous state. The *MDM2* gene encodes for a protein known as murine double minute 2 (MDM2), which functions as a negative regulator of the tumor suppressor protein, p53 (encoded by the *TP53* gene). The MDM2 protein can bind to and degrade p53, leading to decreased levels of this important tumor suppressor protein. Dysregulation of MDM2 can therefore result in increased cell proliferation, decreased apoptosis, and ultimately, cancer development [1,2,3]. Early cell culture studies showed that overexpression of MDM2 in rodent fibroblasts led to increased proliferation and the ability to form tumors when injected into nude mice. This demonstrated that *MDM2* can act as an oncogene, a gene that can promote tumor development [4]. After its initial discovery, the *MDM2* gene was cloned and sequenced. It was found to be located on the long arm of chromosome 12, specifically at the region known as 12q13-14 [5]. It contains two transcriptional promoter elements named P1 and P2. The P2 promoter is dependent on p53. The *MDM2* gene is expressed as different isoforms [6,7,8], and the full-length transcript of *MDM2* encodes a protein of 491 amino acids that plays a critical role [9], where in many human cancers, the MDM2 oncogene is amplified or overexpressed, and high levels of MDM2 are associated with poor prognosis. Under normal conditions, MDM2 is primarily expressed in the nucleus of cells, where it functions as an E3 ubiquitin ligase and regulates the activity of the tumor suppressor protein p53 [6,10]. Apoptosis, DNA repair, and cell cycle arrest can be prevented by MDM2 by binding to and promoting the degradation of p53. However, MDM2 can also translocate from the nucleus to the cytoplasm in response to certain stimuli, such as DNA damage, hypoxia, or oxidative stress. In the cytoplasm, MDM2 can interact with a variety of proteins and play additional roles in regulating cellular processes, such as protein synthesis, RNA splicing, and mitochondrial function. One important function of cytoplasmic MDM2 is the regulation of protein degradation by the proteasome. MDM2 can target proteins for ubiquitination, a process by which a small protein called ubiquitin is added to a target protein, marking it for degradation by the proteasome. In this way, MDM2 can promote the turnover of specific proteins, including itself, under certain conditions.

MDM2 overexpression has been implicated in various processes associated with human and mouse malignant tumors [11], including cell proliferation, DNA damage response, cell cycle regulation, and apoptosis. MDM2 gene amplification [12] or single nucleotide polymorphisms (SNPs) at nucleotide 309 [13,14,15,16,17] are some of the mechanisms which cause increased binding of the transcription factor Sp1 to the *MDM2* promoter, leading to increased transcription and translation of *MDM2* mRNA and protein [18,19]. This is one mechanism that accounts for MDM2 overexpression. In addition to this, the amplification of the *MDM2* gene results in an increase in the number of copies of the gene in the cancer cell genome. In some types of human cancers, particularly solid tumors, overexpression of MDM2 can lead to inactivation of p53 and contribute to tumor progression and resistance to chemotherapy or radiation therapy. Therefore, high levels of MDM2 expression are often associated with poor prognosis in various types of cancer, including breast, lung, bladder, and sarcoma [10,12,20]. This leads to an increase in MDM2 expression levels and can also contribute to the deregulation of p53 signaling pathways.

The protein p53 is the major tumor suppressor protein [21,22,23,24] in vertebrates and has a powerful mechanism to protect from cancer progression [25]. This transcription factor induces cell cycle arrest, apoptosis, or senescence in response to diverse stresses and environmental insults [26,27,28]. Various studies have shown that the p53 pathway plays a crucial role in metabolic homeostasis, stem cell biology, immune microenvironment, and other processes [29]. p53 is constitutively expressed in most normal tissues; however, in normal conditions, MDM2 keeps p53 at a low level due to proteasomal degradation. The remaining p53 activity is further reduced by MDM4 (also known as MDMX), which is another gene that is frequently overexpressed in human cancers and can inhibit the function of p53. As a result of mutations in p53, DNA-specific binding is altered, the protein’s spatial conformation is perturbed, and its thermostabilities are reduced [30].

This negative feedback loop involves p53 promoting the expression of MDM2, which is then responsible for degrading p53 and quenching its activity in the cell [20]. It is estimated that around 50% of human cancers have a mutated form of p53 and over 17% have amplifications of the MDM2 gene; each of these alterations, separately or together, contributes to poor prognosis and treatment failure [10,12,31]. For these reasons, the MDM2–p53 interaction is a target in soft-tissue tumor therapy due to its critical role in regulating the p53 tumor suppressor pathway, which is often deregulated in the *MDM2*-amplified sarcoma cancers.

Many common bone and soft-tissue cancers, including sarcomas, have been shown to be amplified by the MDM2 gene [12]. Soft-tissue tumors are a diverse group of tumors that arise in the connective tissues, including muscles, tendons, cartilage, and fat [32]. The tumors can be benign or malignant, and their clinical presentation and behavior vary widely. Such soft-tissue tumors can cause pain, especially if they are large or compress nearby nerves or other structures, and the malignant soft-tissue tumors are often aggressive and have the potential to metastasize [33].

In many soft-tissue tumors, MDM2 is overexpressed due to gene amplification or other genetic alterations, leading to a decrease in p53 activity and an increase in tumor cell proliferation and survival. For example, MDM2 overexpression has been observed in particular in liposarcomas, which are a type of soft-tissue tumor that arises from fat cells [34,35,36,37]. Moreover, among osteosarcoma patients, MDM2 overexpression correlates with metastasis and advanced stages of the disease and is often associated with more treatment-resistant tumors [38]. Overall, the overexpression of MDM2 in soft-tissue tumors contributes to the aggressive and malignant behavior of these tumors. Therefore, MDM2 has been identified as a potential therapeutic target for the treatment of soft-tissue tumors. Several approaches targeting MDM2, such as small-molecule inhibitors and antisense oligonucleotides, are currently being investigated in preclinical and clinical studies. Research is ongoing to develop drugs that specifically target the MDM2–p53 interaction, with the goal of restoring the function of the p53 pathway and controlling the growth and spread of soft-tissue cancers.

## 2. MDM2 Domains and MDM2 Proteins

### 2.1. MDM2 Domains

MDM2 is a 491 amino acid regulator protein that has multiple domains with specific functions, including binding to p53, localization in the nucleus, exporting from the nucleus, and interactions with other proteins. The N-terminal domain of MDM2 contains a region that interacts with the tumor suppressor protein p53. This domain, also known as the p53-binding domain (PBD), is located in amino acid residues 24-109 of the MDM2 protein and is critical for the regulation of p53 activity. The PBD of MDM2 binds to the transactivation domain of p53, which is located in the C-terminal region of the protein. This interaction leads to the inhibition of p53 activity by promoting its degradation or preventing its transcriptional activity. The interaction between MDM2 and p53 is regulated by a feedback loop in which p53 induces the expression of MDM2, which in turn regulates the activity and stability of p53. This feedback loop plays a critical role in maintaining normal cellular function and preventing the development of cancer.

The NLS domain (residues 181–185) and NES domain are mainly important for transporting the MDM2 protein from the nucleus to the cytoplasm environment and vice versa. In the study by Tao et al., mutations in the NLS of MDM2 prevented it from entering the nucleus and impaired its ability to degrade p53 [39], supporting that MDM2 shuttled p53 from the nucleus to the cytoplasm for its degradation. The cytoplasmic fluorescence (representing p53 export levels) increased in separate studies with the MDM2 NES mutant coexpressed with p53-GFP, and p53 protein levels decreased. As a result of these studies, it has been demonstrated that an intact NES in MDM2 is not required for nuclear export of p53 and that a nuclear-secluded MDM2 cannot trap p53 inside the nucleus.

P53 is degraded by phosphorylating residues within the acidic domain. The RING-finger domain is responsible for its E3 ubiquitin ligase activity, which facilitates the degradation of p53. Furthermore, the C-terminal RING-finger domain can function as an E3 ubiquitin ligase, causing p53 to be ubiquitinated after MDM2 interacts with p53 [40]. Meanwhile, the zinc-finger domain of MDM2 regulates the level of p53 by interacting with ribosomal proteins and inhibiting the degradation of p53 through its acidic domain [41].

### 2.2. MDM2 Proteins, p90 and p76

The human *MDM2* gene is located on chromosome 12q15 and consists of 12 exons that encode the MDM2 protein. The P1 and P2 promoters contain p53 response elements that regulate the expression of two MDM2 proteins, p90-MDM2 and p76-MDM2. The p53-binding domain of P90-MDM2 is fully functional and can bind to p53 at its full length. The P76-MDM2 protein is shorter than the p90-MDM2 protein, and it acts as a negative inhibitor of p90 and activates p53 since it lacks the p53-binding domain [42,43]. The p76-MDM2 protein can also inhibit the function of p90-MDM2 to destabilize p53 [8]. p90 and p76 correspond to exons 3 and 4, respectively.

## 3. MDM2–p53 Interactions

### 3.1. MDM2–p53 Pathway

P53 is an important transcription factor regulating DNA repair, the cell cycle, apoptosis, angiogenesis, and senescence. By interacting with MDM2, it negatively regulates an important defense mechanism that prevents onco-progression. At least 14 different stress signals report information to MDM2 in the MDM2–p53 connection of the pathway [44].

Many small molecules induce post-translational modification of p53 [19,45,46,47]. DNA-damaging agents, including ultraviolet light (UV) radiation [48,49] and cisplatin compounds, can induce phosphorylation-dependent stabilization of p53 [50]. The p53 protein plays important roles in regulating the cell cycle and preventing the development of cancer. The phosphorylation of specific sites on p53, including Ser15, Thr18, and Ser20, modulates the protein’s stability and activity and can affect its interaction with other proteins, including the MDM2 oncoprotein [51,52]. In addition, DNA-dependent acetylation of p53 modification can facilitate chromatin remodeling and activate p53 target gene expression [53,54]. Its C-terminus domain contains these acetylating residues of the p53 protein. The important residues can be acetylated by p300 and cAMP response element-binding (CREB)-binding protein (CBP) [55]. MDM2 can also target these same lysine residues for ubiquitylation [56,57].

The ubiquitin-like protein Nedd8 has been demonstrated to be conjugated to the lysine residues of p53 by the process of either sumoylation or neddylation [58,59,60]. MDM2 can also promote the conjugation of the ubiquitin-like protein Nedd8 to p53 as part of its inhibitory function. The conjugation of Nedd8 with MDM2 can lead to further degradation of p53, thus increasing MDM2’s negative regulation of p53. In a similar way to ubiquitin, Nedd8 binds to lysine residues on target proteins through its C-terminal group [61]. A protein with the F-box domain (FBXO11) inhibits the transcription of the p53 gene by interacting with Nedd8 [62]. This is another way in which the interaction between MDM2 and p53 can influence the stability and activity of p53 and play a role in the regulation of the cell cycle and the prevention of cancer.

Methylation of p53 at lysine residues is a post-translational modification that can influence the stability, localization, and activity of the protein. Methylation of specific lysine residues, such as Lys370, has been shown to stabilize p53, restrict its localization to the nucleus, and enhance the expression of the p21 gene. p21 is a critical downstream target of p53 that functions as a cyclin-dependent kinase inhibitor and can halt the cell cycle and prevent the development of cancer [63]. The mono-methylation of p53 at specific lysine residues, such as K382 by SET8 and K370 by SMYD2, can have opposing effects on the activity of p53 compared to the stabilization effect of methylation mentioned previously. In this case, mono-methylation can attenuate the transcriptional regulation activity of p53, potentially by reducing its ability to bind to DNA or by altering its interaction with other proteins. This can have significant implications for the cell’s response to stress and DNA damage and may contribute to the development of cancer. It highlights the complex interplay of post-translational modifications, such as methylation, in regulating the activity of p53 and determining the outcome of the cell’s response to stress [50,64]. In response to DNA damage, the p53 protein can also be methylated, and this process can affect p53 target gene expression [65]. Overall, the methylation of p53 at lysine residues, along with other post-translational modifications such as acetylation, can play a significant role in regulating the activity of p53 and in determining the cell’s response to cellular stress and DNA damage.

### 3.2. p53 and MDM2 Binding Machinery

Under normal conditions, p53’s level is low because MDM2 is a negative regulator of p53 and acts as a ubiquitin ligase [66,67]. MDM2 binds to the transcriptional activation domain (TAD) region of p53, which is amphipathic in nature. This process inhibits p53’s activity by preventing the interaction between p53 and other proteins. Thus, necessary steps for the activation of p53-mediated transcription are blocked. This binding of MDM2 to p53 is an important mechanism for maintaining low levels of p53 in the cell because excess p53 can lead to cell cycle arrest or cell death.

In the N-terminal p53-binding domain of MDM2, the binding pocket is hydrophobic [68]. The TAD region of p53 can have hydrophobic amino acid residues on the hydrophobic side. Phe19, Trp23, and Leu26 play essential roles between the p53 and MDM2 interactions [68]. After the hydrophobic α-helix structure is formed, the binding pocket of MDM2 moves close to the structure and forms the p53–MDM2 complex through hydrogen bonding. By ubiquitinating p53’s N-terminal activation domain, MDM2 inhibits transcriptional activity and proteasomal degradation. The cyclin-dependent kinase (CDK) inhibitor p21 is one of the p53 target genes. It is a negative regulator of the cell cycle [20]. In mutated p53 cells, there is no p21 activation, which results in the progression of the cell cycle even with damaged DNA [69].

### 3.3. MDM2 Has Important Roles Independent of p53

**Figure 1:**
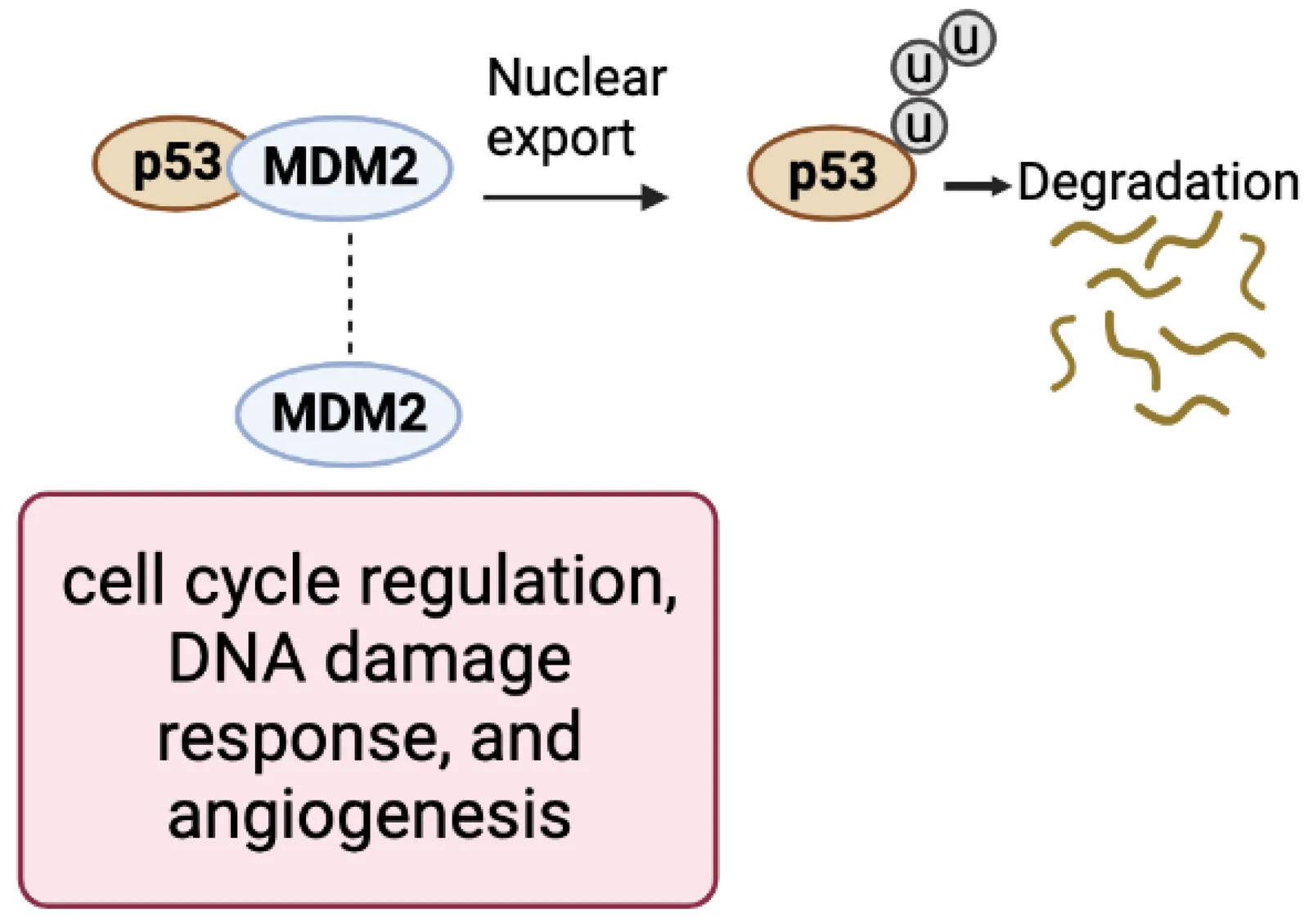
MDM2: p53-independent activities. In addition to negatively regulating p53 tumor suppressor protein, MDM2 also regulates a number of other cellular proteins and is involved in multiple processes of cancer progression independent of p53. A number of oncogenic proteins can be stabilized and activated by MDM2, as well as cellular processes such as angiogenesis and DNA damage response.

Both clinical and preclinical evidence suggests that MDM2 has important roles in the cell, independent of p53 (Figure 1). MDM2 has been shown to regulate the stability and activity of a number of oncogenic proteins, such as RAS, MYC, and Cyclin E, and to play essential roles in cellular processes, including cell cycle regulation, DNA damage response, and angiogenesis. Additionally, MDM2 can act as an oncogene itself by promoting tumor cell survival and promoting cell growth and proliferation.

MDM2 can drive cell cycle progression by affecting the cellular process factors independent of p53. For example, MDM2 can affect cellular processes such as DNA synthesis and DNA repair by interaction with DNA polymerase [70,71,72], dihydrofolate reductase (DHFR) [73], centrosome amplification [74], and the MRN DNA complex containing Nbs1 [74,75]. A number of proteins, including retinoblastoma (Rb) and the transcription factor E2F-1 complex, interact with MDM2 [76,77]. These processes are independent of p53, and drive cell cycle progression (typically S-phase).

MDM2 can inhibit apoptosis, which is also independent of p53. MDM2 has been demonstrated to interact with the E2F1/Rb pathway to inhibit apoptosis [78]. In addition to the neddylation of p73, MDM2 plays an anti-apoptotic role by preventing p53 transactivation and mediating p73 neddylation [76,78]. MDM2 affects both pro-apoptotic as well as anti-apoptotic proteins since MDM2 also upregulates the translation of anti-apoptotic XIAP (X-Linked Inhibitor of Apoptosis), thus inactivating caspase-mediated apoptosis [79]. Overall, these suggest that, in addition to being a negative regulator of p53, MDM2 also affects the functions of other cellular proteins.

## 4. Targeting and Potential Targeting MDM2 for Soft-Tissue Tumors

### 4.1. Soft-Tissue Tumors—Liposarcoma and Desmoid Tumors

In some types of cancer, such as liposarcoma and osteosarcoma, the *MDM2* gene can become amplified, leading to an overproduction of the MDM2 protein. The *MDM2* amplification represents the hallmark of well-differentiated liposarcoma/atypical lipomatous tumor, dedifferentiated liposarcoma, intimal sarcoma, as well as low-grade osteosarcoma. This overproduction can inactivate wild-type p53, leading to the formation and progression of cancer [80], making inhibition of the MDM2 protein a logical target to support p53’s suppressive activity [81].

Liposarcomas are a type of cancer that originate from fat cells and are relatively rare, accounting for about 1% of all sarcomas. They are typically found in the soft tissue of the body, particularly in the extremities, such as the arms and legs, as well as the retroperitoneum, which is the area behind the peritoneum in the abdomen. Liposarcomas can be classified into several subtypes based on their histological appearance, including well-differentiated, myxoid, round cell, and dedifferentiated liposarcoma. Each subtype has distinct clinical and pathological characteristics, and the treatment and prognosis can vary depending on the subtype. It is important to diagnose liposarcomas early and accurately, as they can often be mistaken for benign fatty tumors.

*MDM2* amplification is one of the key diagnostic markers for liposarcoma [34,35,36], and it is often used to differentiate it from other types of sarcomas. For example, the well-differentiated liposarcoma/atypical lipomatous tumor (WDL/ATL) is a type of tumor which is often seen in middle-aged adults [82,83]. It is important to know where it is because it can be dangerous if it is in certain places. Typical lipomatous tumors are lumps that can typically be removed by surgery and are usually found in the arms and legs. Well-differentiated liposarcomas are lumps that are often more difficult to remove and can be found in the abdomen, around the testicles. Sometimes a tumor can have extra copies of certain genes, both of which contain multiple copies of *MDM2*. The amplification results in nuclear MDM2 protein overexpression. Other molecular markers, such as TP53 mutations and CDK4 amplification, are also commonly found in liposarcoma and can be used for diagnosis and prognosis. Co-amplifications of CDK4, GLI1, and HMGA2 are common in the 12q14-15 region, but *MDM2* is the main driver. The use of molecular markers in the diagnosis of liposarcoma can improve the accuracy of the diagnosis and help guide treatment decisions. Immunohistochemistry and FISH for MDM2 are tests used to help doctors diagnose WDL/ATL [84,85].

**Figure 2:**
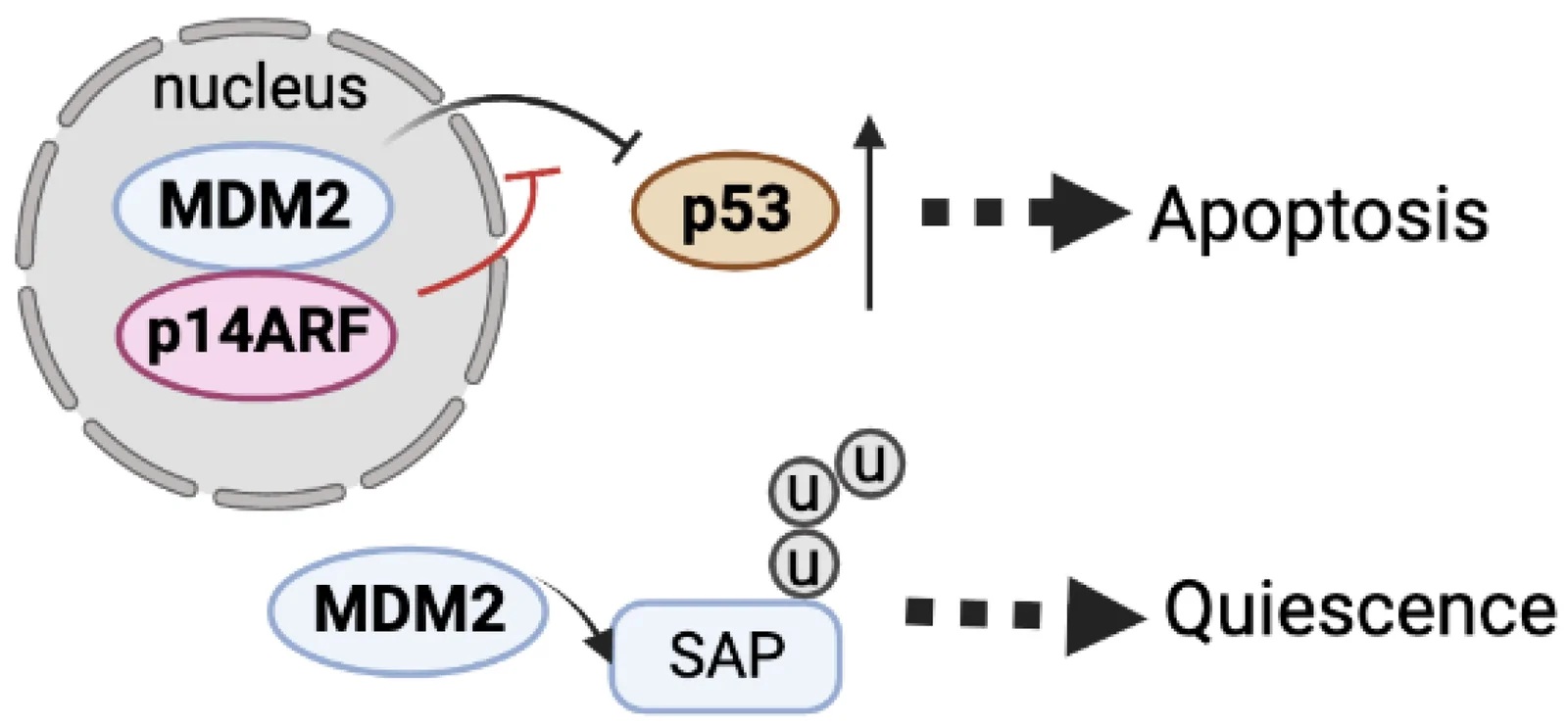
Schematic representation of p14ARF epigenetic silencing represents a valuable target for affecting MDM2 function in sarcoma. As a key regulator of p53 and a direct inhibitor of MDM2, p14ARF is encoded by the partially open reading frame p14ARF located on the CDKN2A locus. P14ARF binds to MDM2 at its N-terminus, sequestering it inside the nucleus. MDM2 is negatively regulated by it, as it binds to it and prevents it from degrading p53.

The p14ARF epigenetic silencing event represents a valuable target that can affect MDM2 function in sarcoma. p14ARF is encoded from the partially open reading frame p14ARF located on the CDKN2A locus and acts as a key regulator of the tumor suppressor p53. It is a direct inhibitor of MDM2 [86,87], since the N-terminal domain of p14ARF binds to MDM2 which sequesters the MDM2 shuttle to the nucleus [88,89]. This process negatively regulates the MDM2 protein by p14ARF binding to it and preventing MDM2’s ability to degrade p53 (Figure 2). Myxoid and pleomorphic sarcomas are associated with epigenetic alterations in p14ARF. Davidović et al. demonstrated that an epigenetic silencing occurred through the methylation of the promoter region of p14ARF in myxoid and pleomorphic sarcoma [90]. Subsequently, in their cohort of round cell liposarcoma samples, Oda et al. found hypermethylation of the p14ARF promoter, resulting in reduced p14ARF protein expression and overexpression of p53 [91]. It is demonstrated that variable levels of p14ARF expression are observed in dedifferentiated liposarcoma (DDLPS) [92]. Epigenetic silencing of the p14ARF gene can occur due to changes in the regulation of gene expression without altering the DNA sequence itself, resulting in the loss of its function and the subsequent activation of MDM2. This leads to the degradation of p53 and the formation and progression of cancer, including sarcoma. Overall, p14ARF epigenetic silencing is a valuable target which can affect MDM2 function in sarcoma. Targeting p14ARF epigenetic silencing in sarcoma has been proposed as a potential strategy for restoring p53 function and inhibiting MDM2 activity. This could lead to improved treatment outcomes for sarcoma and other cancers in which p14ARF and MDM2 play a role.

Reducing MDM2 in cells can drive them into a more stable senescent state, and this effect has been observed in several types of cancer, including well-differentiated and dedifferentiated liposarcoma, breast cancer, lung cancer, and glioma. Marta Kovatcheva et al. suggests that the proteolytic turnover of MDM2, a protein that is often overexpressed in cancer, is required for CDK4i-induced senescence [93]. The study also highlights the role of the E3 ligase activity of MDM2 and the expression of ATRX as new regulators of geroconversion, the process by which quiescent cells become senescent [93]. As part of senescence, cancer cells are prevented from proliferating and malignant progression is slowed. Kovatcheva et al. demonstrated that the loss of MDM2 accompanies and drives geroconversion, or the transition of cells to senescence [93]. It appears that MDM2 directly ubiquitinates a senescence-activating protein to maintain cell quiescence in non-responder cells after being knocked down by MDM2 (SAP, Figure 2). Overall, these suggest that the *MDM2* gene can not only become amplified, which is a hallmark of well-differentiated liposarcoma/atypical lipomatous tumor, but also can be targeted treatment outcomes for sarcoma and other cancers.

Desmoid tumors are a type of soft-tissue tumor that originate from fibrous tissue. There is no direct study about the association between desmoid tumors and MDM2; however, our lab recently obtained a human desmoid tumor cell line with loss-of-function screening using an shRNA pool and we found some essential genes that are important for desmoid tumor cell growth. *MDM2* is one of the genes that we found; by inhibiting MDM2 in human desmoid tumors, it decreases the proliferation of desmoid tumor cancer cells. There is some non-direct evidence that targeting MDM2, or its specific downstream target, making MDM2 a potential therapy for human desmoid tumors. For example, the role of p53 in desmoid tumor patients was investigated, and the data show the overexpression of p53, and Ki-67 indicates a high probability of recurrence [94]. Overall, these data suggest that MDM2 is also important for the progression of desmoid tumors.

### 4.2. Strategies to Target the MDM2–p53 Interaction for Soft-Tissue Tumors

The MDM2–p53 interaction can be targeted in two ways: by blocking the expression of MDM2 and by inhibiting the binding of MDM2 to p53. Inhibition of the MDM2 oncoprotein can limit its interaction with p53, thus preventing p53 degradation and leading to higher levels of p53. Gene silencing techniques have already proved the effectiveness of this approach. Activation of p53 may also be achieved by inhibiting MDM2–p53 binding. Since protein–protein interactions normally involve large and flat surfaces, targeting them with small molecules is still challenging because low molecular weight compounds are hard to disrupt.

**Figure 3:**
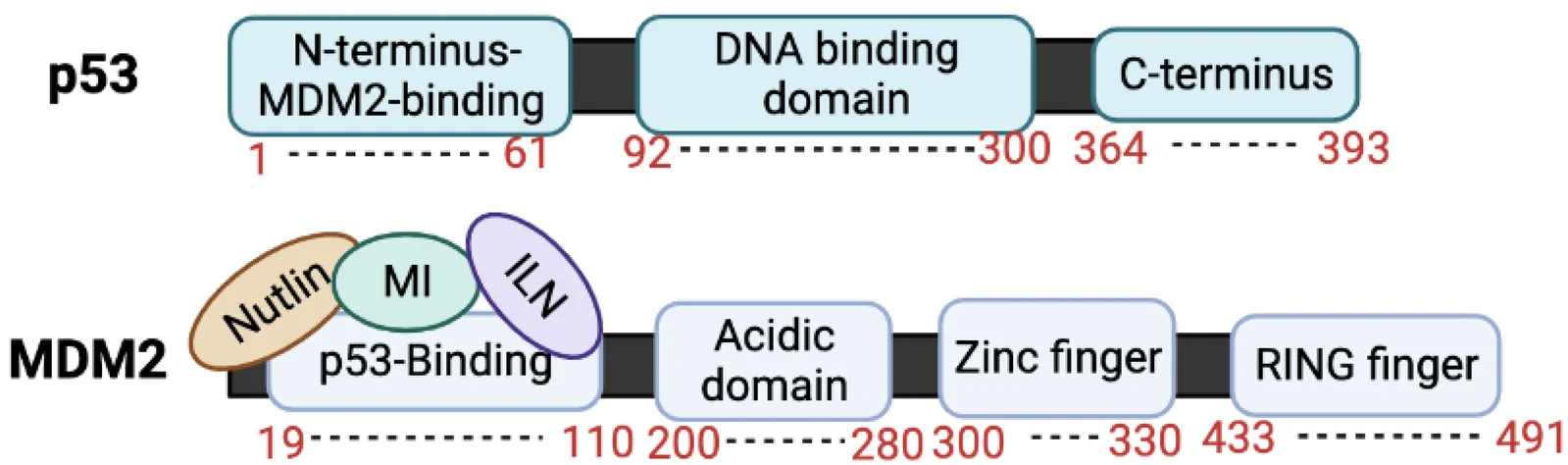
Schematic representation of the MDM2 and p53 proteins and the binding areas for small-molecule inhibitors. Examples of small-molecule inhibitors that target the MDM2 protein include nutlin, cis-imidazoline; MI, spiro-oxindoles; and ILN, isoindolinone. By binding to specific areas of the MDM2 protein, these small-molecule inhibitors can effectively inhibit its function, leading to an increase in p53 levels and a reduction in tumor growth.

A hydrophobic pocket on the surface of MDM2 binds Phe19, Trp23, and Leu26 of p53, which are crucial to its binding [95,96,97]. Thus, the protein architecture provides a framework to design small molecules that mimic the interaction. Several small-molecule inhibitors, such as nutlins [96,97,98,99], ILN (isolindones) [100], MI (spiro-oxindoles) [101], and chalcone derivatives, have been developed via combinatorial library screening based on this principle (Figure 3) [101,102].

Nutlins have been extensively studied in preclinical models. Nutlins are a class of small-molecule inhibitors of MDM2 that have shown promise in preclinical studies for cancer therapy. Nutlins bind to the p53-binding pocket of MDM2, thereby preventing the MDM2–p53 interaction and leading to the stabilization and activation of p53. One advantage of nutlins is their specificity for MDM2, which allows for selective targeting of cancer cells that overexpress MDM2 while sparing normal cells. Nutlin-3a, a protein that activates caspases upon apoptosis, activated apoptosis in 45% of MDM2-amplified osteosarcoma cell lines after 48 h, according to Vassilyev et al. [103]. Similarly, Nutlin-3 treatment of in vivo mouse xenograft models bearing wild-type p53 osteosarcoma cell lines resulted in 90% inhibition of tumor growth.

Recent studies have shown improved potency and selectivity of HDM201, a new MDM2 inhibitor. Using HDM201 treatment methods, Jeay et al. showed preclinical evidence that p53 wild-type osteosarcoma cells with *MDM2* amplified growth inhibition [104]. A significant increase in P21 mRNA expression was observed after HDM201 treatment in vitro, which encodes for a potent cyclin-dependent kinase inhibitor that plays a crucial role in cell cycle arrest associated with DNA damage [105,106,107,108].

BI 907828 is a MDM2–p53 antagonist which inhibits the interaction between the p53 and MDM2 proteins [109]. *MDM2* gene amplification has been found in nearly all cases of dedifferentiated liposarcoma [109,110,111]. BI 907828 significantly inhibited tumor growth in two mouse models of DDLPS harboring amplifications of MDM2, compared with doxorubicin and control groups. In light of these results, BI 907828 was tested in clinical settings [112]. The monotherapy study with BI 907828 had treated 107 patients, including 39 with DDLPS, by July 2022. Phase Ia/Ib study results showed an 88.9% disease control rate (DCR) for 36 patients evaluable for DDLPS and 8.1 months as the median progression-free survival (PFS) (range 0.8–21.0 months; 13 patients were still receiving treatment). The preliminary studies as well as a phase Ia/Ib clinical trial of BI 907828 in patients with solid tumors, especially those with dedifferentiated liposarcoma, have shown promising results.

The compounds that are now under clinical evaluation (and summarized in Table 1) may have clinical potential, and the recent results are obtained from their assessment.

MDM2-targeting antisense oligonucleotides (ASOs) have been explored as potential cancer therapies. This test oligonucleotide, Oligo AS (5′UGACACCTGTTCT-CACUCAC-3′), has previously been described [113] and has been evaluated for antitumor activity in human colon cancer models both in vitro and in vivo. Anti-MDM2 antisense oligonucleotides can be developed in the future as cancer therapeutic agents by themselves or in combination with conventional chemotherapeutics, based on the findings of this study.

## 5. Conclusions

Targeting the MDM2–p53 interaction has emerged as a promising strategy for soft-tissue tumor therapy, as it allows for the restoration of p53 function and the induction of tumor cell death. Several approaches are being explored for this purpose, including small-molecule inhibitors of MDM2, antisense oligonucleotides, and peptides that disrupt the MDM2–p53 interaction.

In addition, other strategies are also being investigated to reactivate p53 in cancer cells, such as targeting other negative regulators of p53, such as MDM4, or activating upstream signaling pathways that lead to p53 activation. Ultimately, the success of these strategies will depend on their ability to selectively target soft-tissue tumor cells while sparing normal cells as well as their efficacy in clinical trials.

## Figures and Tables

**Table 1:** Clinical trials with MDM2 inhibitors in soft-tissue tumors and other cancers.

Compound	Clinical Study	Status
BI 907828	Compare BI 907828 With Doxorubicin in People with a Type of Cancer Called Dedifferentiated Liposarcoma	Recruiting
Navtemadlin	Navtemadlin and Radiation Therapy in Treating Patients with Soft-Tissue Sarcoma	Active, not recruiting
DS-3032b	Ascending Dose and Exploratory Expansion Study of DS-3032b, an Oral MDM2 Inhibitor, in Subjects with Relapsed and/or Refractory Multiple Myeloma	Terminated
Milademetan	Safety, Tolerability, and Pharmacokinetics of Milademetan Alone and with 5-Azacitidine (AZA) in Acute Myelogenous Leukemia (AML) or High-Risk Myelodysplastic Syndrome (MDS)	Terminated
Cytarabine; Milademetan Tosylate	Milademetan Tosylate and Low-Dose Cytarabine with or Without Venetoclax in Treating Participants With Recurrent or Refractory Acute Myeloid Leukemia	Completed
APG-115	APG-115 in Salivary Gland Cancer Trial	Recruiting
ALRN-6924	A Study of ALRN-6924 for the Prevention of Chemotherapy-induced Side Effects (Chemoprotection)	Terminated
HDM201	Trametinib + HDM201 in CRC Patients With RAS/RAF Mutant and TP53 Wild-type Advanced/Metastatic Colorectal Cancer Mutant and TP53 Wild-type	Recruiting
KRT-232	Safety and Efficacy of KRT-232 in Combination with Acalabrutinib in Subjects With R/R DLBCL or R/R CLL	Recruiting
LXS196	A Phase I Study of LXS196 in Patients with Metastatic Uveal Melanoma	Terminated

Data from ClinicalTrials.gov. National Library of Medicine: http://www.clinicaltrials.gov (accessed on 13 April 2023).

## Data Availability

Not applicable.
