# Correction: Sun, S.Y. MDM2 Implications for Potential Molecular Pathogenic Therapies of Soft-Tissue Tumors. *J. Clin. Med.* 2023, *12*, 3638

**DOI:** 10.3390/jcm15135287

**Published:** 2026-07-07

**Authors:** Sylvia Yao Sun

**Affiliations:** Sarcoma Biology Laboratory, Department of Surgery, Memorial Sloan Kettering Cancer Center, 417 E 618 St, New York, NY 10065, USA; sy1viaysun5@gmail.com

## Error in Funding

In the original publication [1], the funder was: Dr. Aimee Crago’s grant: MSKCC NIH Cancer Center Grant P30-CA008748; NIH/NCI R37-CA241856; Kristen Ann Carr Foundation. The updated Funding information should include: This research received no external funding.

## Removal of an Author

In the original publication [1], Aimee Crago has been removed from the authorship, as she was included in the original publication without her consent. Accordingly, her affiliation has also been removed.

The author states that the scientific conclusions are unaffected. This correction was approved by the Academic Editor. The original publication has also been updated.
